# Follow-up Visits and Changes in Pain Scores Reported by Oncology Outpatients After Initial Presentation With Severe Pain

**DOI:** 10.7759/cureus.965

**Published:** 2017-01-09

**Authors:** Brett Hill, Dwight Moulin, Michael Sanatani

**Affiliations:** 1 Medical Oncology, Schulich School of Medicine & Dentistry, London Regional Cancer Program, Western University, London, Ontario, CA; 2 Neuro-oncology, Schulich School of Medicine & Dentistry, London Regional Cancer Program, Western University, London, Ontario, CA

**Keywords:** cancer pain, symptom management, follow-up

## Abstract

**Background:**

In addition to tumour treatment, the management of symptoms such as pain is an important component of cancer care. Pain management is a complex field and prior studies have highlighted many different clinical care responses to a cancer patient presenting with severe pain. We explored follow-up and how pain screening scores changed over time, among a cohort of cancer outpatients, and how follow-up was scheduled after the initial visit.

**Methods:**

The care provided to 96 patients seen at the London Regional Cancer Program was reviewed for the 12-week period following presentation with severe pain >7/10. Follow-up ESAS (Edmonton Symptom Assessment System) scores, visits, and compliance were documented.

**Results:**

Follow-up ESAS data was available for 41/96 patients. Mean ESAS pain decreased from 8.4/10 to 3.6/10 among those patients with follow-up; however, for 55/96 patients, no follow-up ESAS score was available (deceased n=3, no follow-up visit n=41, no pain score reported, n=11).

**Conclusions:**

Despite a very high proportion of documented active pain management plans in the case of cancer patients presenting with severe pain, very little follow-up directed specifically at pain management was performed. Cancer treatment appears to be the primary determinant of oncology follow-up timing at our centre.

## Introduction

Pain is one of the most prevalent symptoms in patients with metastatic cancer and has wide-ranging deleterious effects on activity, mood, and sleep, to name a few [1]. Quality of life is compromised when pain control is poor [1]. However, pain is commonly mismanaged and inadequately treated in a clinical setting [2-6]. Yennurajalingham et al. found that 50% of patients surveyed, who were experiencing moderate to severe pain, reported no pain relief following analgesia, and 32% of patients with moderate pain indicated an increase in pain. These findings have been replicated throughout the literature [2-6], indicating that work needs to be done to better understand the management of cancer pain.

Addressing some of the concerns surrounding pain management has been difficult, and the various components of the pain management process need to be defined and improved [7-9]. Assessment of pain is the first step of effective pain management. At the London Regional Cancer Program (LRCP) in Ontario, Canada, all patient visits include electronic completion of the Edmonton Symptom Assessment System (ESAS) which includes a pain scale (zero to 10). The completed form can be seen on the patient’s electronic record by the nurse and physician prior to the clinic visit [10-11]. ESAS is a standardized symptom screening tool used to assess common symptoms in cancer patients: pain, tiredness, nausea, depression, anxiety, drowsiness, appetite, well-being, and shortness of breath, and a blank space for the patient to fill in any additional problem [10]. Zero represents absence of the symptom, while a score of ten represents the worst severity possible. A pain score of greater than or equal to seven is considered to be severe [10]. A decrease in two points or greater has been suggested as being clinically significant [12], while inadequate pain management is defined as no clinically significant change or an increase in pain score over time.

In a previous study at the LRCP by Sanatani et al., a high proportion (83%) of patients who reported severe pain (ESAS >7), received an active intervention targeted at managing that pain [11]. Further study is needed to determine the subsequent effectiveness of various care approaches in improving the pain level that is initially reported. The next step is to investigate the changes, if any, over time after the initial visit where a high pain score was reported by the patient. This current study examines the timeline of the ESAS pain subscale changes over three months following the initial visit, to assess ongoing attention to pain management and any correlation with other ESAS subscales.

Informed consent was obtained from patients, and IRB approval was provided by Western University Health Sciences Research Ethics Board (approval #106326).

## Materials and methods

### Purpose and study endpoints

The purpose of the present study was to determine the mean changes in ESAS pain score from baseline, occurring at follow-up visits after the implementation of various pain management regimens in patients presenting with severe pain (7-10/10). The follow-up pain scores were recorded for the visits as listed in Table 1. Associated data collected from the charts included information on whether the previously documented management plans actually were implemented, baseline demographics, and the other ESAS subscales (all rated 1-10): nausea, well-being, depression, anxiety, dyspnea, drowsiness, fatigue, and anorexia. In addition, the number of patients for whom follow-up ESAS scores were available was determined in order to assess follow-up appointment scheduling.

**Table 1:** Pain score assessment times

*Baseline*
*First return visit with ESAS data available, 14 weeks after baseline visit*
*First return visit with ESAS data available, 4–8 weeks after baseline visit*
*First return visit with ESAS data available, 8–12 weeks after baseline visit*
*Latest return with ESAS data available, before 12 weeks from baseline*

### Eligibility and patient selection

A retrospective chart review was done (both electronic and paper contents) on the patients previously selected and reported on by Sanatani et al [11]. The patient medical record (both electronic and paper contents) was reviewed for these sequential unique patient visits, starting December 15, 2011, at solid tumour medical, surgical, and radiation oncology clinics at the LRCP, with documented patient pain levels >7/10 at the electronic kiosk prior to seeing the nurse and physician. Patient visit identification was done by a search of the electronic hospital records, starting with the first visit registered at our centre at 8:00 a.m. on December 15, 2011, and searching forward, screening first by severe ESAS pain level, and then by inclusion and exclusion criteria. Patients attending only the palliative care or pain management clinics were excluded from the current study because, by definition, patients attending these clinics had been specifically referred for review of an active pain management plan. Follow-up visits for administration of chemotherapy or radiation without physician involvement, urgent visits to the ambulatory care bay for dehydration, transfusions, electrolyte imbalances, or other acute issues were excluded.

## Results

Ninety-six charts were found eligible for this follow-up study and were reviewed (both electronic record and paper chart including nursing documentation). Baseline characteristics are indicated in Table 2. Out of these patients, 41 had at least one follow-up ESAS score documented within 12 weeks of the baseline visit. Forty-four patients had no follow-up visit within 12 weeks of the baseline visit, and the reasons are outlined in Table 3.

**Table 2:** Baseline characteristics

Recorded Data		
Age (mean, range)	63, 26-95	
Gender (M/F)	38/58	
		n
Assessed cause of pain at baseline visit (n)	Cancer related	40
	Treatment related	21
	Non-cancer related	23
	Unknown	12
Primary tumour site (n)	Breast	20
	Colorectal	10
	Prostate	8
	Renal	4
	Bladder	2
	Uterine	2
	Cervix	1
	Pancreatic	1
	Head and Neck	10
	Neuroendocrine	13
	Peritoneal	2
	Skin	2
	Gallbladder	2
	Unknown Primary	3
	Brain	1
	Lung	15
Medical specialty seeing patient at baseline visit (n)	Medical Oncology	58
	Radiation Oncology	33
	Surgical Oncology	5
Treatment intent (n)	Curative: 45 / Non-curative: 51

**Table 3:** Reasons for absence of follow-up ESAS scores (n=55)

Reason	n
Deceased	3
No follow-up booked at LRCP within 12 weeks	37
Did not attend booked follow-up	4
Follow-up visit within 12 weeks but ESAS not completed	11

**Figure 1:**
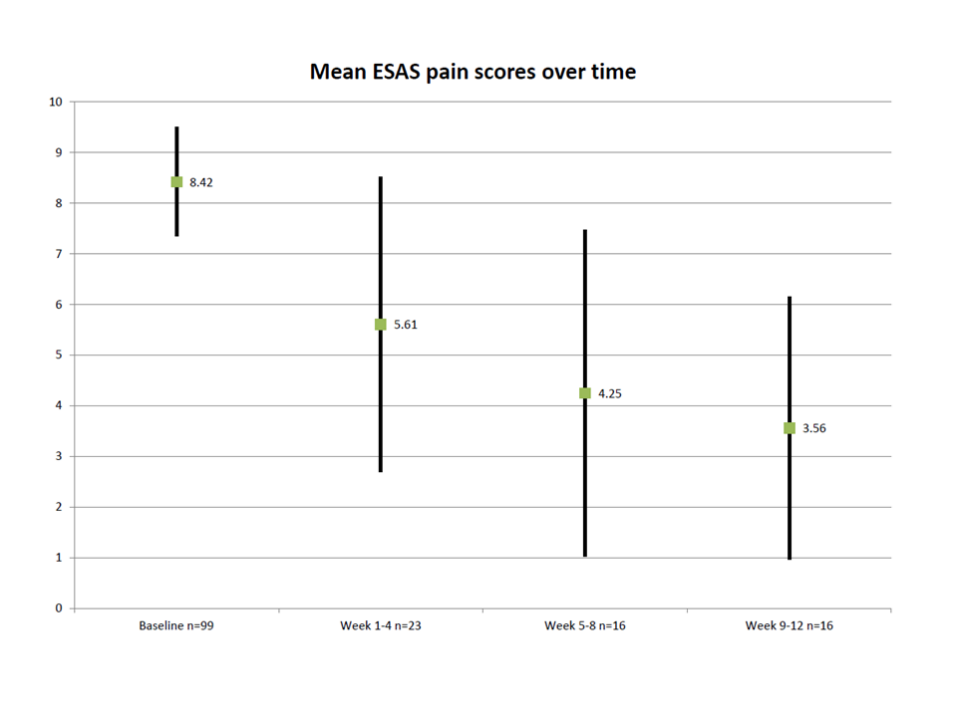
Changes in ESAS pain scores over time

The changes in ESAS pain scores for those patient visits captured within the 12-week follow-up window are indicated in Table 4 and Figure 1. Over time, the mean ESAS pain score from visits in the three follow-up periods (1-4 weeks, 5-8 weeks, and 9-12 weeks after initial visit) decreased (Figure 1). However, the numbers of patients with follow-up visit ESAS scores declined, and there was a correlation between the last ESAS score recorded and the length of interval between the baseline and the last ESAS score (r=0.379, p=0.015). Situations where the initially planned pain management was not carried out are summarized in Table 5.

**Table 4:** Pain scores over time

ESAS Pain Scores					
	Baseline	Follow-up between weeks 1-4	Follow-up between weeks 5-8	Follow-up between weeks 9-12	Longest follow-up visit available
n	96	23	16	16	41
Mean ESAS (SD)	8.43 (1.08)	5.61 (2.98)	4.25 (3.34)	3.56 (2.68)	4.78 (3.29)
Mean ESAS change from baseline (SD)	n/a	2.96 (2.82)	4.25 (3.61)	4.69 (2.70)	3.59 (3.24)
Median time from baseline visit (days)	n/a	13.0	37.5	69.0	42.0

**Table 5:** Discrepancies between original pain management plan and actual care (n=16)

Discrepancy	n
Patient did not take or stopped prescribed medication – lack of perceived effect	2
Patient did not take or stopped prescribed medication – adverse effect, ran out, or took other medication instead	4
Unforeseen acute event (fall, hospital admission)	2
Patient declined recommended treatment or consultation	5
Treatment plan changed by physician	2
Pain resolved	1

Associations between patient characteristics and the change in ESAS pain score from baseline to longest follow-up were examined. No significant predictive factors were found.

## Discussion

In this follow-up study, 96 patient charts were reviewed to assess the changes in pain scores reported by cancer patients after a baseline visit where they had indicated their pain was severe (>7/10). As documented in our earlier study [11], the severe pain was explicitly addressed by the oncology team with an active pain management plan in a relatively high proportion of patient visits (83% of all visits, including 97% of visits where the pain was deemed cancer- or treatment-related, and 57% of visits where the documented cause of pain was non-cancer related or unknown). In the majority of cases, these plans were carried out, and if they were not, it was usually due to patient preferences. However, an unexpected finding was the low number of visits that were scheduled within a short timeframe, which would have allowed reassessment of the pain scores.

In fact, the majority of patients’ first follow-up visits were not captured in this study, as they fell outside the three-month window after the baseline visit was reviewed. Follow-up visit scheduling did not appear to be short-term, as 37 out of 96 patients received their next visit three months or later after baseline. At our centre, at the time of this study, follow-up visit timing was entirely at the discretion of the oncologist, and an electronic follow-up booking order, set with prepopulated, recommended follow-up timeframes, was not in use yet (as it is now). Therefore, the timing of the booked follow-up visit entirely reflects the conscious clinical decision of the oncologist. We therefore conclude that despite the initial attention given to the severe pain, in-clinic follow-up was often not arranged primarily to reassess pain levels in the short-term but was perhaps linked more to the expected progression of the underlying cancer.

The limiting factor in generalization of the results of this study is the relatively small number of patients actually seen in follow-up. It is unknown what occurred in many patients with respect to their pain management as a large proportion received no follow-up within three months after severe pain was documented on the ESAS. Nevertheless, for those patients for which follow-up data is available, pain documented by the ESAS decreased from baseline at each subsequent interval over the 12-week period. No associations were discovered between other ESAS scores and a change in pain level over 12 weeks. Similarly, Hwang et al. [13] described that no independent predictors of pain relief were identified over a three-week longitudinal study. These findings confirm the complex nature of pain and relate to difficulties faced in management across modalities. There was a correlation between length of time from baseline to last ESAS pain level. With increasing length of time from baseline, pain severity as rated by the ESAS decreased. The improvement in pain level as documented on the ESAS may be confounded by passage of time as opposed to a therapeutic response to the pain management plan.

Previous studies have shown similar findings in that pain ratings from admission to follow-up were decreased, and this reduction was not associated with treatment [14]. Deardoff et al.[14] proposed that at baseline, when patients are being evaluated for treatment, subjective pain level on a rating scale is inflated. When treatment was already initiated or completed and obtaining treatment was no longer a concern, patients may more accurately rate their pain. Paice [15] proposed that patients under-report their symptoms over time, as they do not want to burden their family or physician.

## Conclusions

In this study of oncology follow-up after an initial patient presentation with severe pain, we found that only a minority of patients had oncology follow-up booked within three months. This leads to the conclusion that despite pain having been addressed at the initial visit, this assessment did not necessarily lead to follow-up in a timely manner to specifically reassess the pain. In a subset of the 96 cancer patients initially reporting severe pain, reduction in pain was observed over time, independent of several variables including type of pain and type of treatment. However, this finding was limited by small numbers available for follow-up. This study illustrates that pain level reassessment itself did not appear to be the main determinant of booking follow-up. Given the physical constraints of a typical cancer centre, perhaps alternative forms of support and follow-up, other than a clinic visit, could be considered when assessing a patient with severe pain. This could take the form of timed telephone calls, mail survey follow-up, or more precise communication to the primary healthcare providers with regard to pain management follow-up steps.
